# Supplementary material for: Leukemia stemness and co-occurring mutations drive resistance to IDH inhibitors in acute myeloid leukemia
Source: Nat Commun. 2021 May 10;12:2607. doi: 10.1038/s41467-021-22874-x (PMC8110775; doi:10.1038/s41467-021-22874-x)
Supplement: Supplementary file 1 — Supplementary Information [file 41467_2021_22874_MOESM1_ESM.pdf]

Supplemental Appendix

Supplement to: Wang F, Morita K, DiNardo C, et al. **Leukemia stemness and co-occurring mutations drive resistance to IDH inhibitors in acute myeloid leukemia**

Contents

Figure S1.
Figure S2.
Figure S3.
Figure S4.
Figure S5.
Figure S6.
Figure S7.
Figure S8.
Figure S9.
Figure S10.
Figure S11.
Figure S12.
Figure S13.
Figure S14.
Table S1.
Table S2.

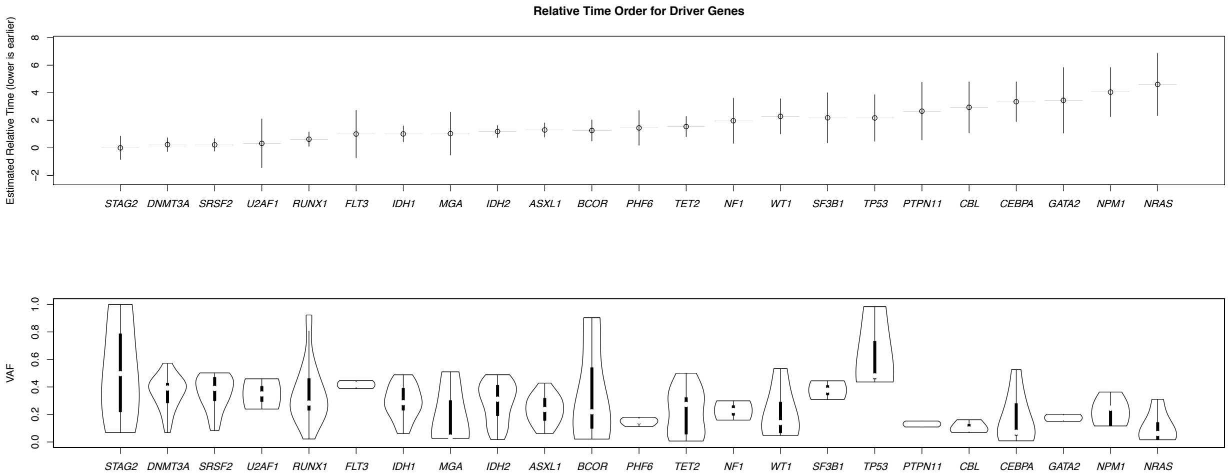

Figure S1. Relative timing of the mutation accrual in the IDHi-treated AML patients. Forrest plots (top) showing the relative timing of mutation accrual for each driver genes detected in the baseline samples (N=54) of the IDHi-treated AML patients. The dots (center of the error bars) represent the ability estimates based on an unstructured Bradley-Terry model. The lower the ability estimates, the earlier the relative timing would be. The error bars represent the standard errors. Violin plots (bottom) showing the driver mutation VAF distributions for all driver genes. Source data are provided as a Source Data file.

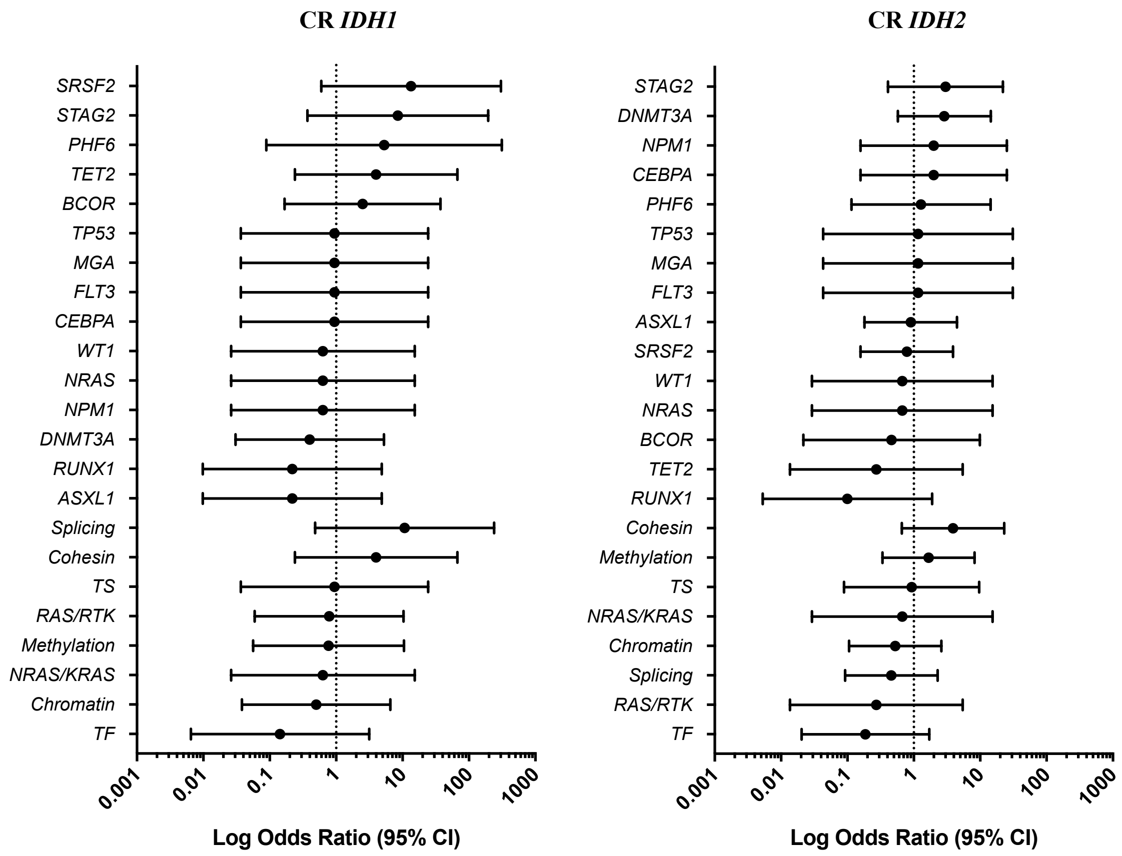

Figure S2. Forrest plots showing enrichment of the mutations at baseline against Complete Remission (CR) by logarithmic odds ratio for *IDH1*- (left; N=21) and *IDH2*- (right; N=38) mutated patients. Circles (center of the error bars) represent odds ratios. The error bars represent 95% confidence interval of odds ratio. Source data are provided as a Source Data file.

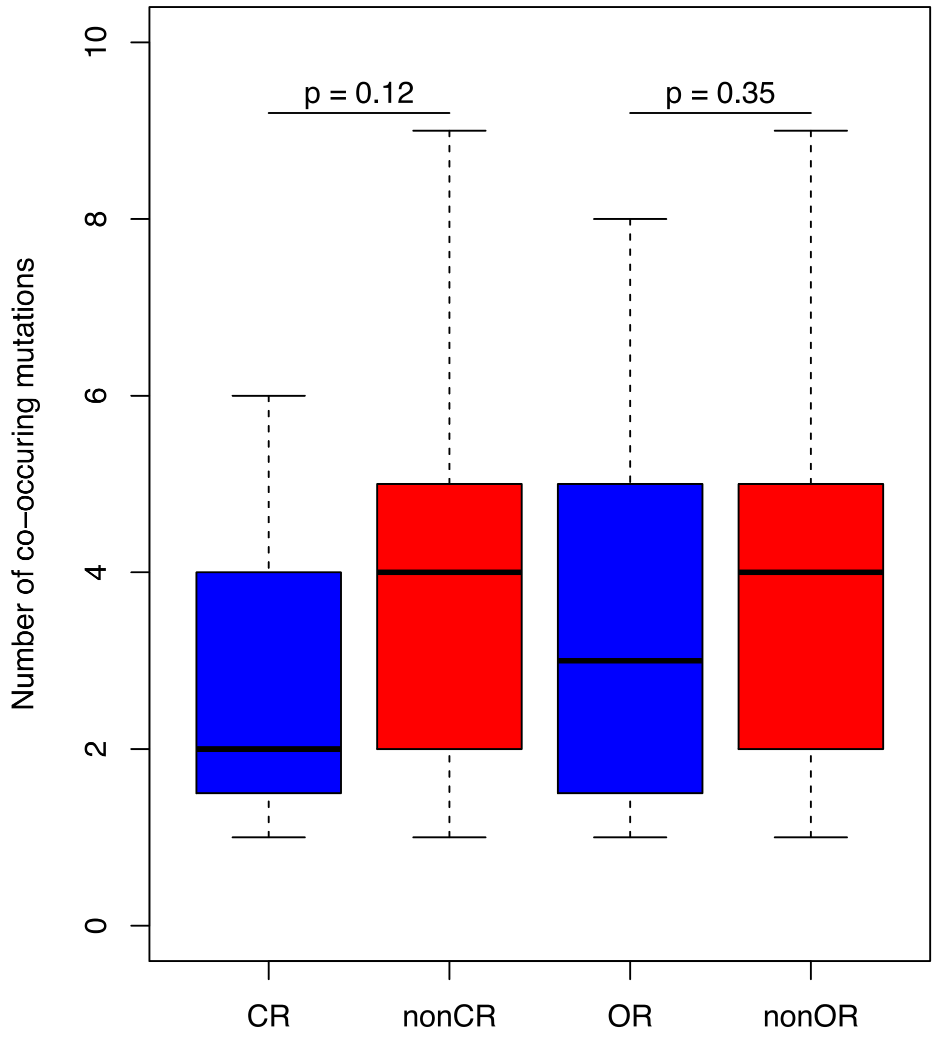

Figure S3. The number of co-occurring mutations at baseline did not have a significant impact with respect to the clinical response in our cohort. Box plots comparing the number of co-occurring mutations in baseline samples from patients who achieved CR (N=11), non-CR (N=44), OR (N=28) or non-OR (N=27). Abbreviations: CR, complete remission; OR, overall response. Two-sided Student's t-test was performed. Box plot shows the minimum, first quartile (Q1), median, third quartile (Q3), and maximum. Source data are provided as a Source Data file.

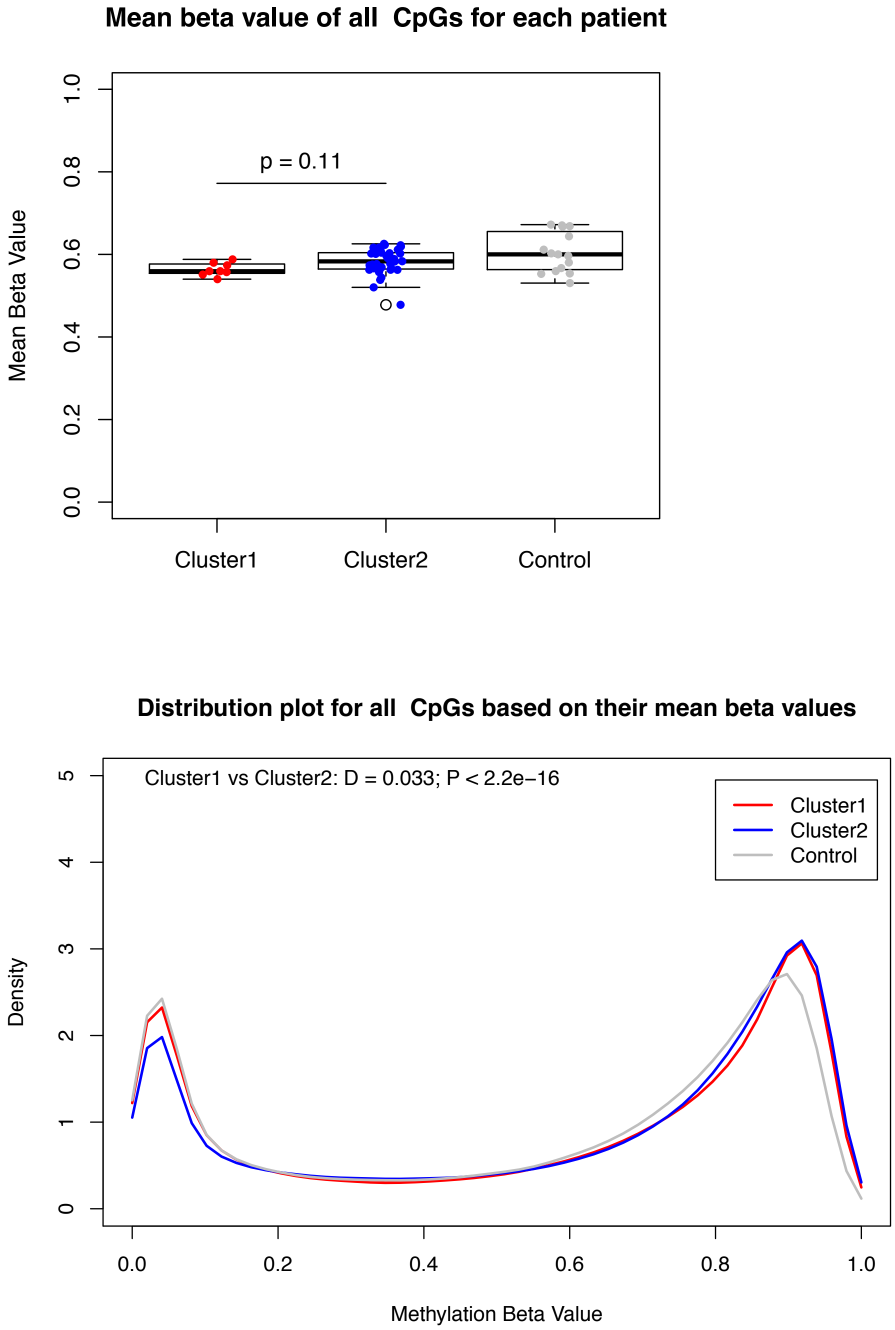

Figure S4. Cluster 2 patients show hypermethylation at baseline time point when compared to Cluster 1 patients. (Top) Box plot comparing mean methylation beta value of all CpGs among Cluster 1 baseline (N=36) and Cluster 2 baseline (N=15) samples. *IDH1/2* wild type AML samples (N=8) are used as control. Box plot shows the minimum, first quartile (Q1), median, third quartile (Q3), and maximum. Two-sided Student's t-test was performed. (Bottom) Density distribution of all CpG probes with methylation beta values comparing Cluster 1 baseline and Cluster 2 samples. Two-sided Kolmogorov–Smirnov test was performed. *IDH1/2* wild type AML samples (N=8) are used as control. Source data are provided as a Source Data file.

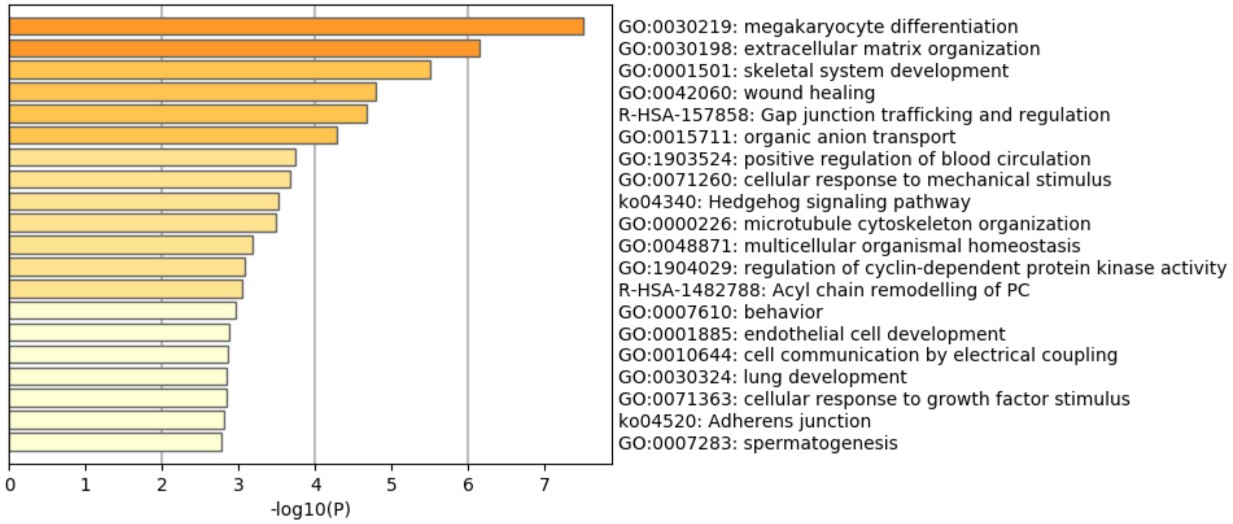

Figure S5. Metascape analysis of downregulated genes which were associated with hypermethylated promoter DMPs in Cluster 2. Source data are provided as a Source Data file.

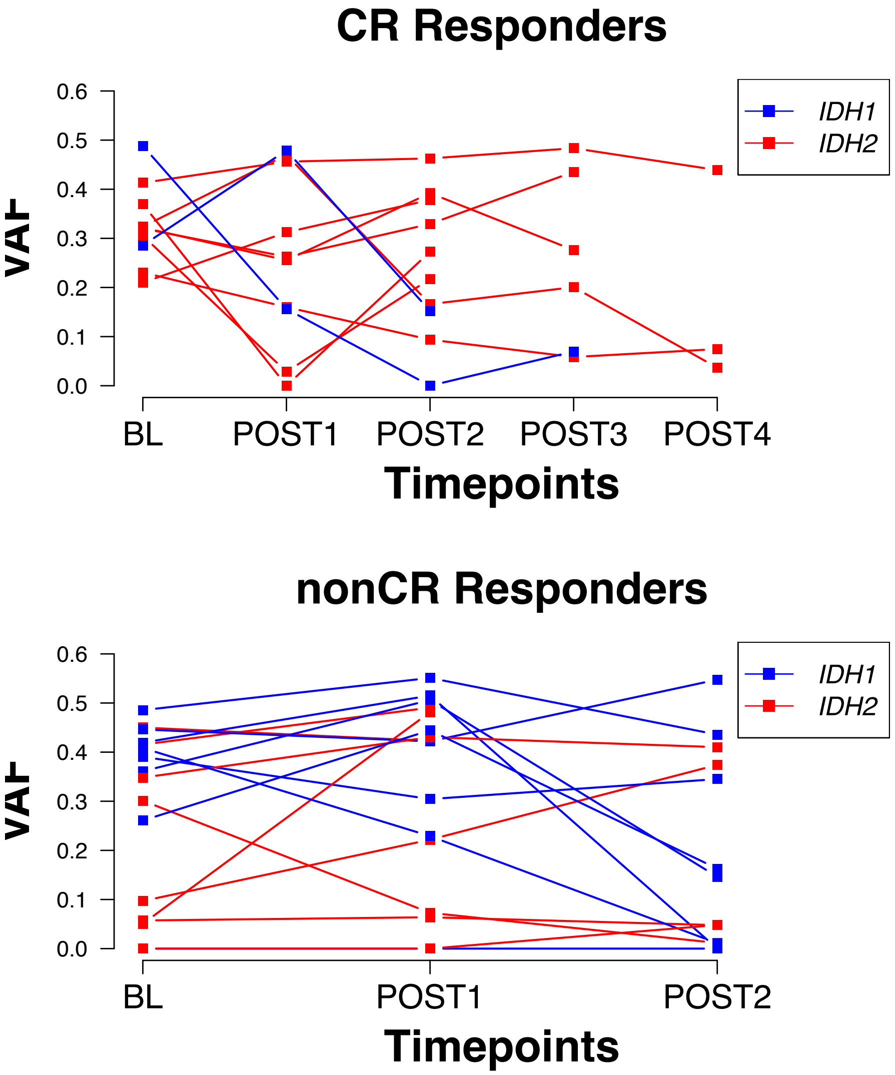

Figure S6. Longitudinal trend of VAF for *IDH1/2* mutations for responders achieving CR (top) and all other non-CR (bottom) responders. Abbreviations: CR, complete remission; VAF, variant allele frequency; BL, baseline; POST, post-treatment. Source data are provided as a Source Data file.

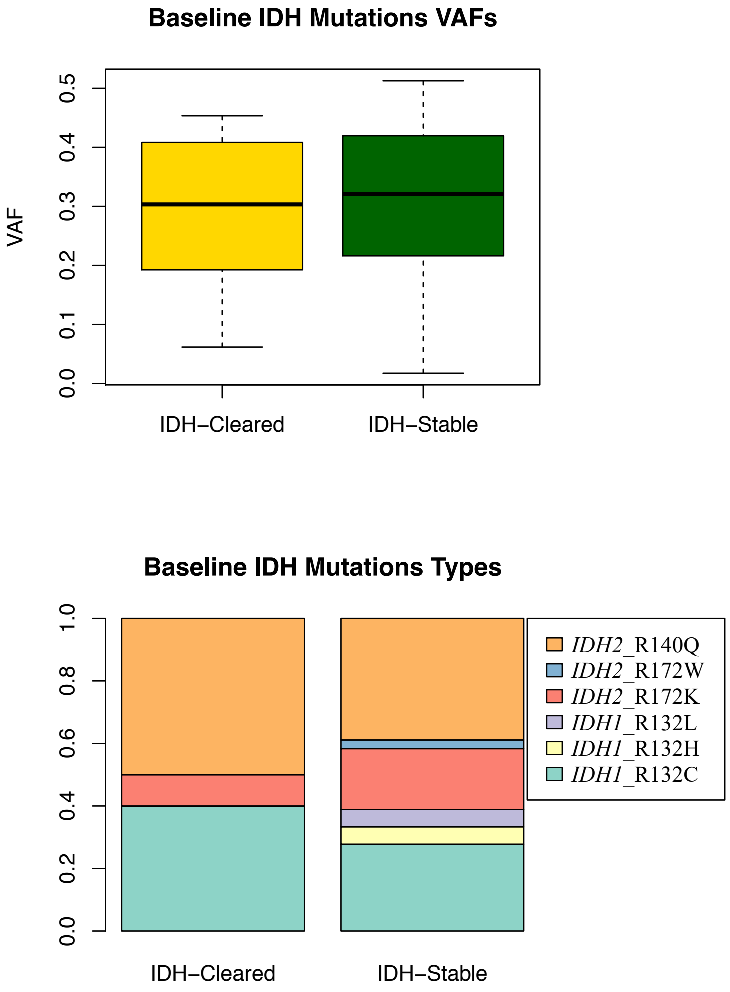

Figure S7. The baseline VAF or the types of *IDH* mutations did not predict the clearance of the mutations. Box plots (top) showing the VAF distribution of *IDH* mutations in *IDH*-Cleared and *IDH*-Stable baseline samples. Box plot shows the minimum, first quartile (Q1), median, third quartile (Q3), and maximum. Bar plots (bottom) showing the mutation-type distribution in *IDH*-Cleared and *IDH*-Stable baseline samples. Source data are provided as a Source Data file.

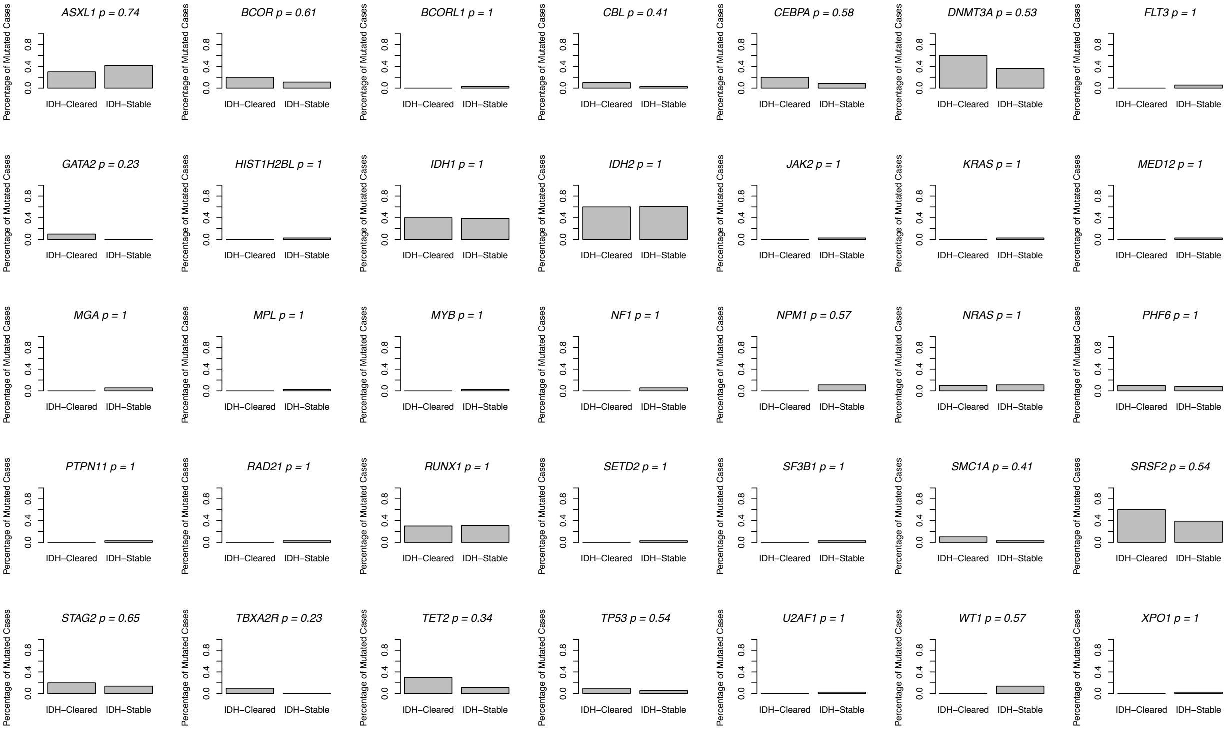

Figure S8. No correlation between *IDH* mutation clearance and the co-occurring mutations. Bar plots showing the mutated case percentage in *IDH*-Cleared (N=10) and *IDH*-Stable (N=36) baseline samples for each co-mutated gene. Two-sided Fisher's exact test was performed. Source data are provided as a Source Data file.

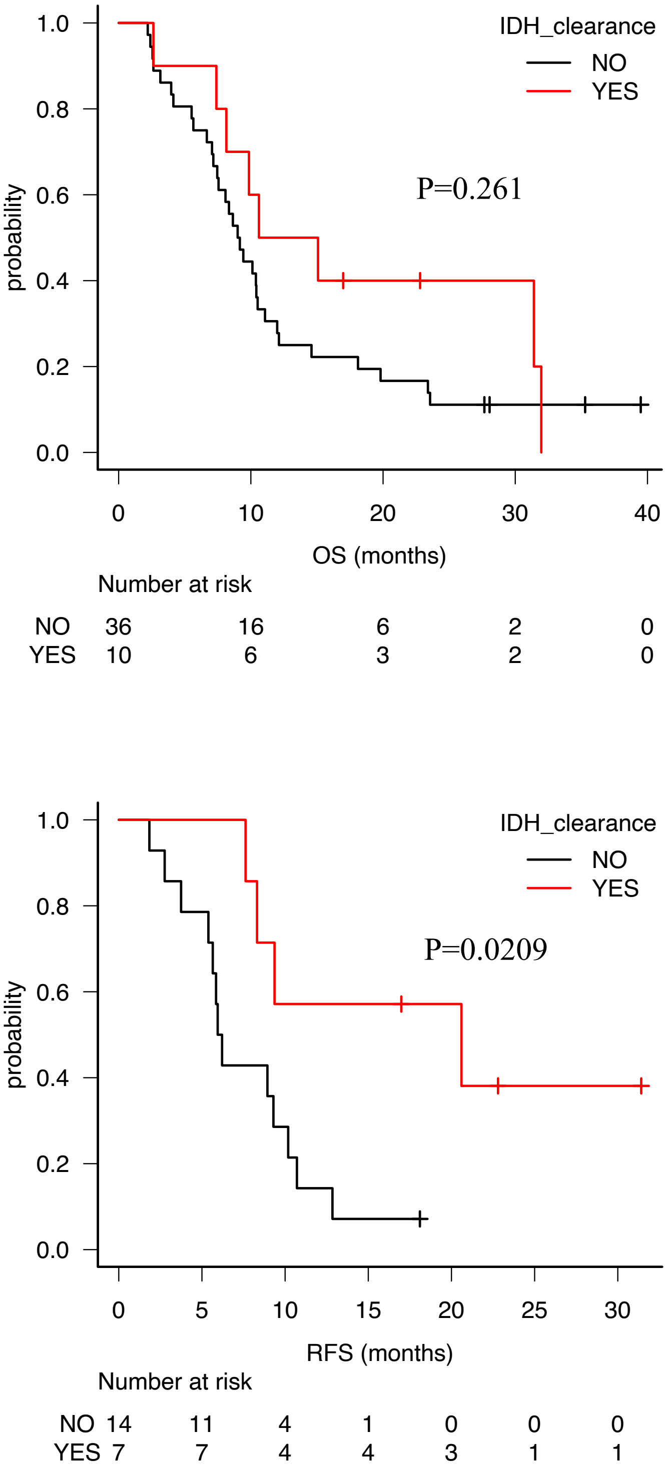

Figure S9. Overall survival (OS, top) and Relapse-free survival (RFS, bottom) comparing *IDH*-Cleared and *IDH*-Stable patients. Two-sided Log-rank test was performed. Source data are provided as a Source Data file.

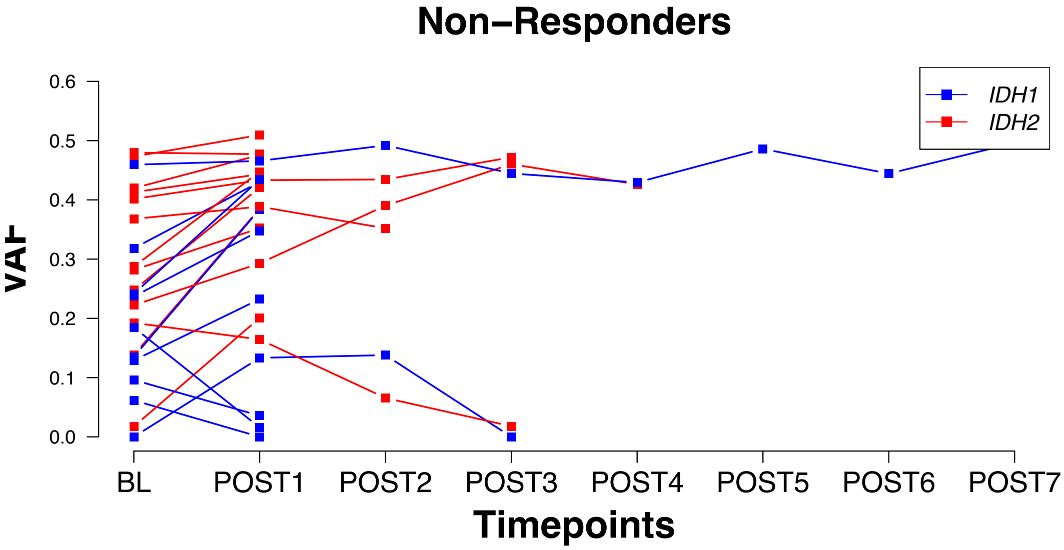

Figure S10. Longitudinal trend of VAF for *IDH1*/*2* mutations for non-responders. Abbreviations: VAF, variant allele frequency; BL, baseline; POST, post-treatment. Source data are provided as a Source Data file.

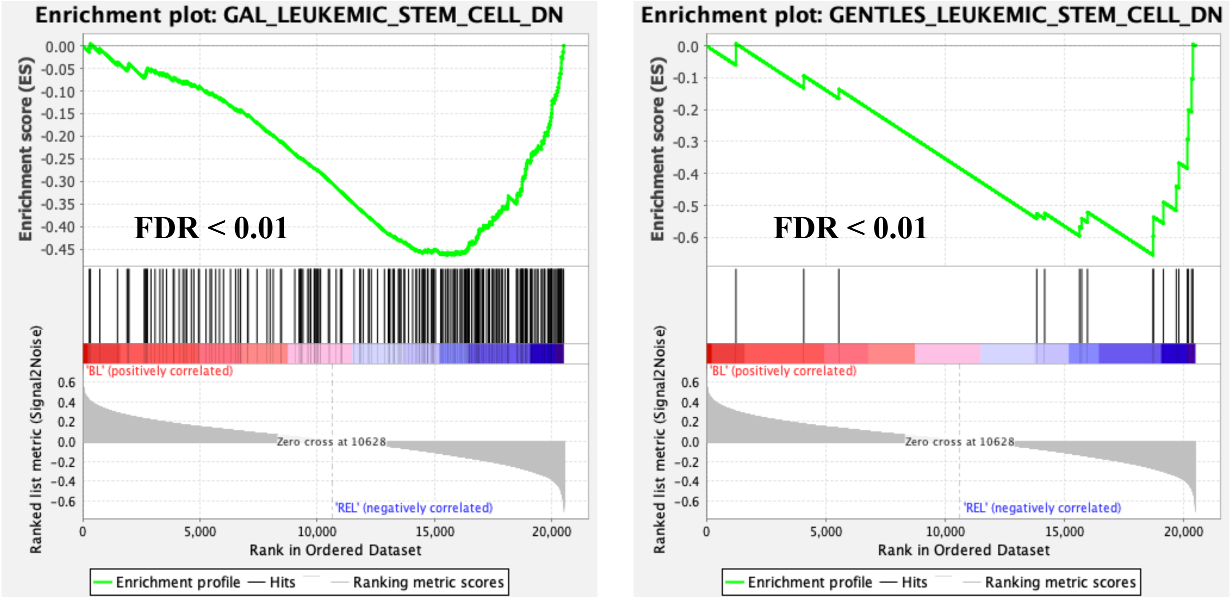

Figure S11. Gene Set Enrichment Analysis comparing gene expression profiles between baseline and relapse samples. Genes downregulated in leukemia stem cells (LSC) are enriched in relapse samples. Source data are provided as a Source Data file.

UPI2370759

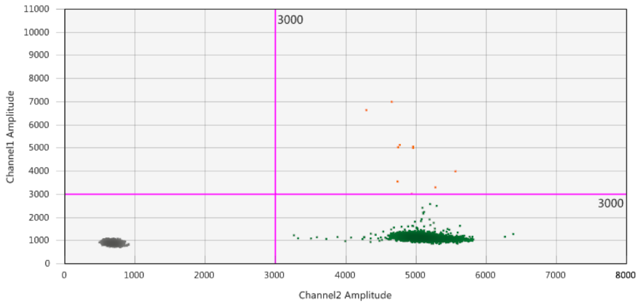

Positive control

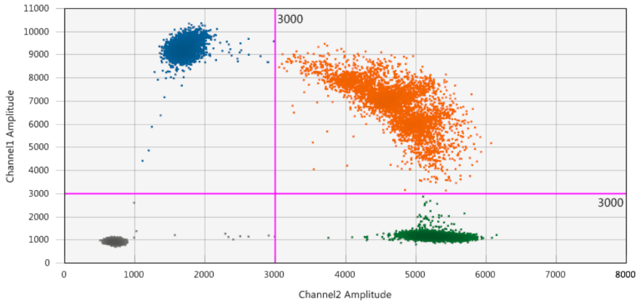

Negative control

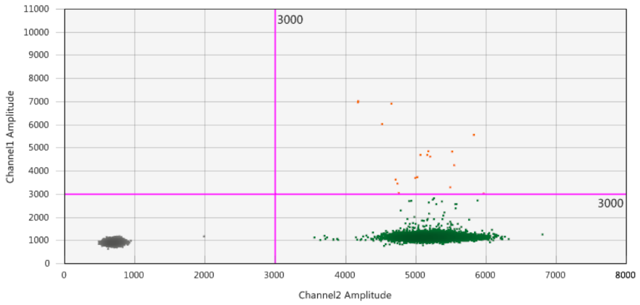

No template control

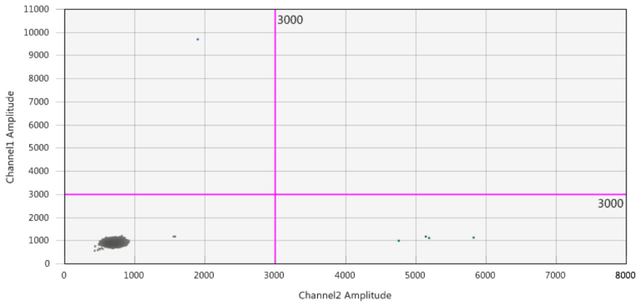

Figure S12. ddPCR did not detect *IDH1* R132C in UPI2370759. 2-D plots showing amplitude in two channels. Blue cluster shown in upper-left quadrant represents droplets with mutant DNA only. Orange cluster shown in upper-right quadrant represents droplets with both mutant and wildtype DNA. Green cluster shown in lower-right quadrant represents droplets with wildtype DNA only. Grey cluster shown in lower-left quadrant represents droplets without DNA from targeted locus. Fractional abundance was 0.0714% in UPI2370759.

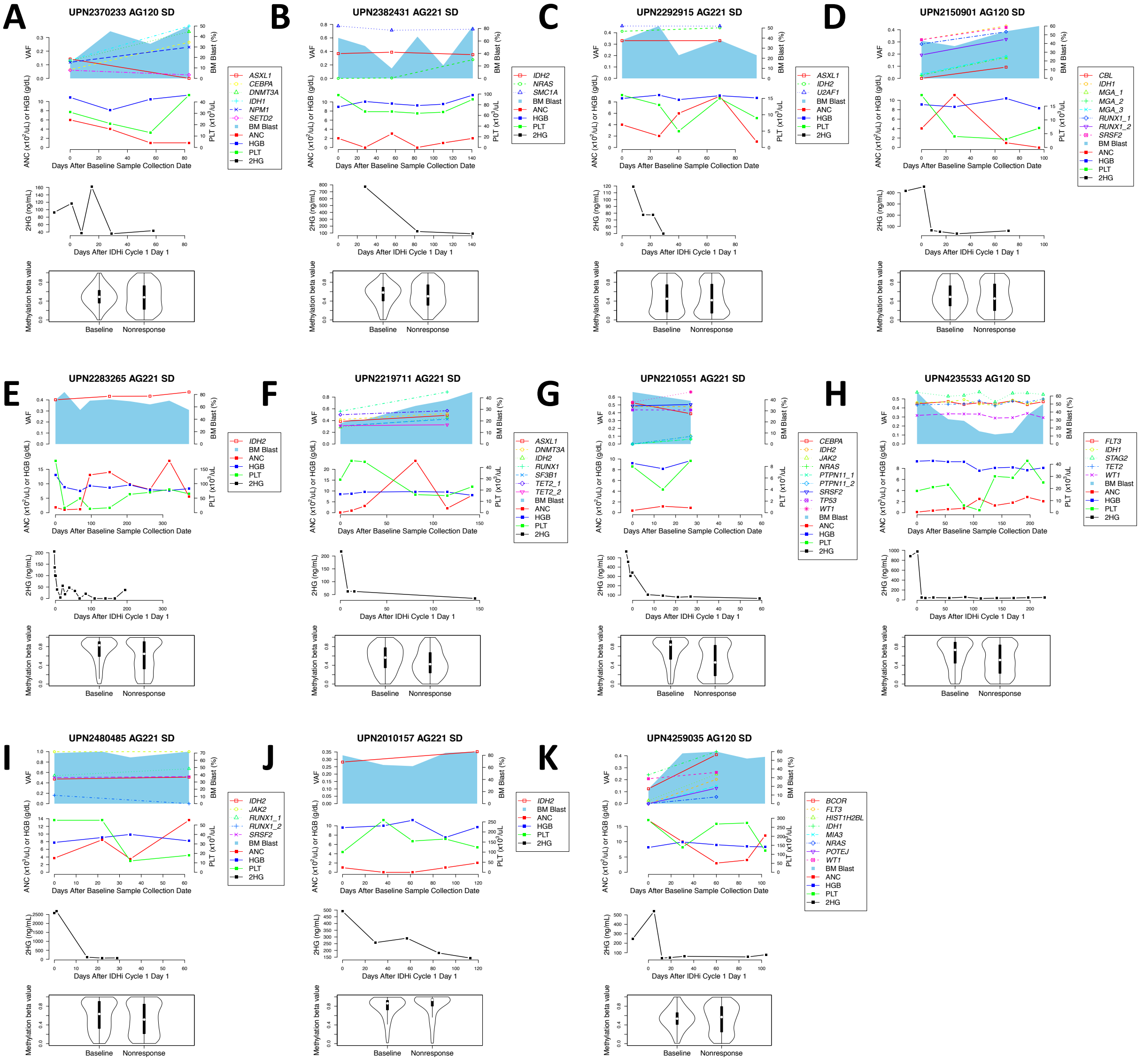

Figure S13. Genetic and epigenetic co-evolution in 11 non-responders. (**A-I**) Cases that show co-suppression of 2HG and DNA methylation after IDH inhibitor. (**J-K**) Cases that show 2HG suppression but without demethylation. Violin plot shows the minimum, first quartile (Q1), median, third quartile (Q3), and maximum. Source data are provided as a Source Data file.

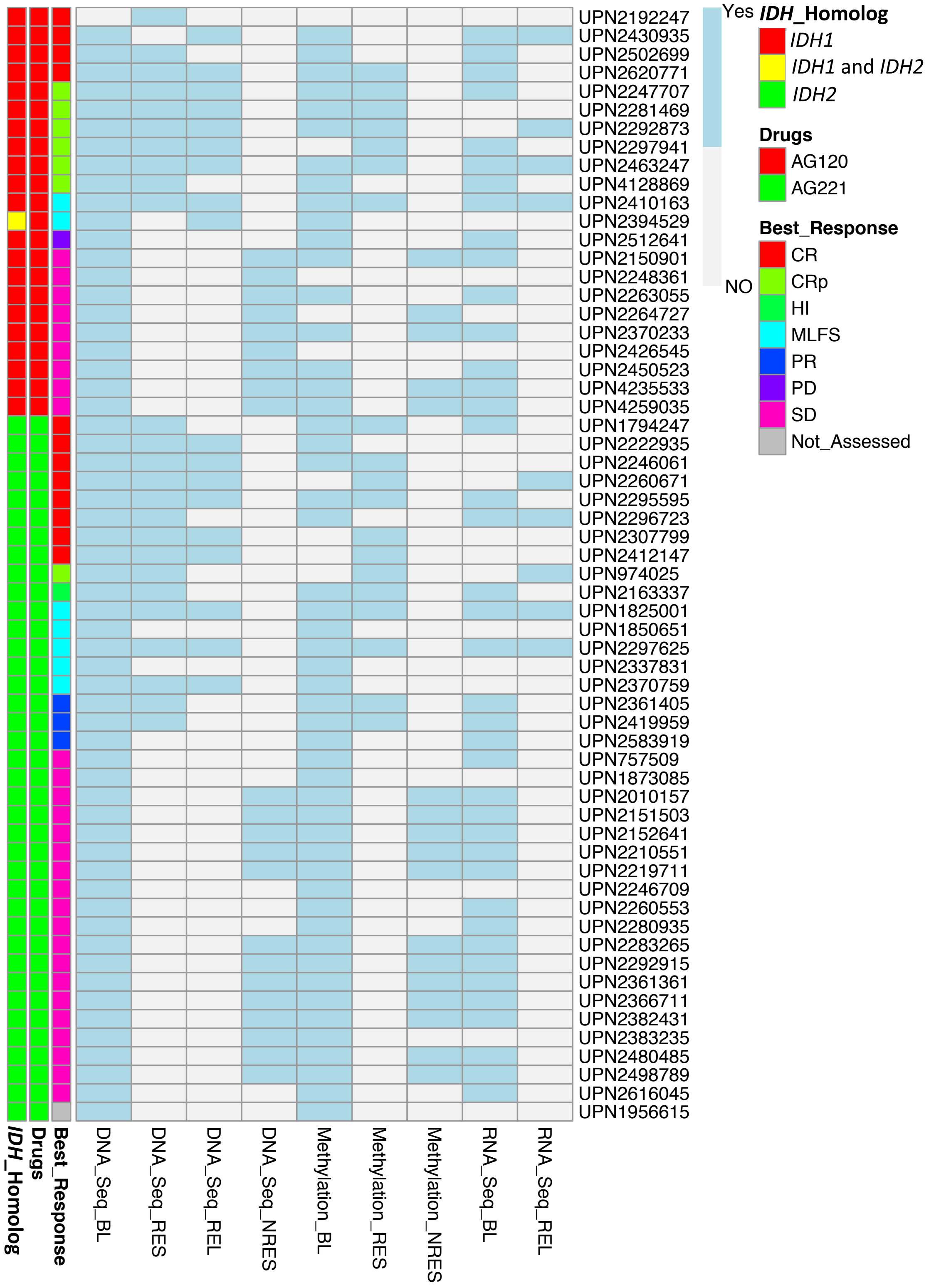

Figure S14. Summary heatmap for sample availability across all analytic platforms for all 60 patients. Abbreviations: BL, baseline; RES, response; REL, relapse; NRES, non-response. Source data are provided as a Source Data file.

Table S1. Comparison of clinical features between included and excluded patients.

	included (N=60)		excluded (N=51)		
	median	IQR	median	IQR	p
WBC	1.5	0.9-2.4	1.6	1.0-7.2	0.241
ANC	0.2	0.1-0.6	0.4	0.2-2.1	0.035
HGB	9.3	8.8-10.0	9.4	8.8-10.2	0.328
PLT	33	20-57	29	16-66	0.461
BM blast, %	41	19-64	28	9-53	0.051
Age	72	60-77	66	55-73	0.007
	No.	%	No.	%	
Karyotype					0.671
intermediate	40	69	38	75	
poor	18	31	13	26	
Sex					0.56
female	25	42	18	35	
male	35	58	33	65	
Drug					0.004
AG120	22	37	33	65	
AG221	38	63	18	35	
Best Response					
CR	12	20	3	6	
CRp	7	12	4	8	
MLFS	7	12	4	8	
PR	3	5	2	4	
HI	0	0	2	4	
SD	28	47	19	37	
PD	1	2	6	12	
Not done	1	2	11	22	
Responder	30	51	15	38	0.221
Non responder	29	49	25	63	

ND – Two-sided Student's t-test; non-ND – Two-sided Mann–Whitney U test
WBC – non-ND; ANC– non-ND; HGB – ND; PLT– non-ND; BM blast, %– non-ND; Age– non-ND

Abbreviations: ND, normal distribution
Source data are provided as a Source Data file.

Table S2. List of 295 genes targeted by next generation sequencing.

ABCC9	*CALR*	*CUL5*	*FANCD2*	*HIST1H2BF*	*LEF1*	*NBN*	*PLA2G2D*	*SF3B1*	*TINF2 (TIN2)*
ABL1	*CARD11*	*CUX1*	*FANCE*	*HIST1H3D*	*LRP1B*	*NCOR1*	*PLCG2*	*SFRS1*	*TLR2*
ACTG1	*CBL*	*CYLD*	*FANCG*	*HIST1H4D*	*LTB*	*NCOR2*	*POT1*	*SFRS7*	*TLR9*
AKT1	*CBLB*	*DAXX*	*FANCI*	*HNRNPK*	*LUC7L2*	*NF1*	*POU2AF1*	*SGK1*	*TNFAIP3*
ANKRD11	*CCND1*	*DCLRE1C*	*FANCL*	*HRAS*	*LYN*	*NFE2*	*PRDM1*	*SH2B3*	*TNFRSF14*
ARID1A	*CCND3*	*DDX3X*	*FAS*	*ICOS*	*MALT1*	*NFKB1*	*PRKCB*	*SHH*	*TNKS*
ARID1B	*CD200*	*DIS3*	*FAT1*	*ID3*	*MAP2K1*	*NFKB2*	*PTEN*	*SMAD2*	*TOX*
ARID2	*CD274*	*DKC1*	*FAT3*	*IDH1*	*MAPK1*	*NFKBIA*	*PTPN1*	*SMC1A*	*TP53*
ARID5B	*CD58*	*DLC1*	*FBXW7*	*IDH2*	*MAX*	*NFKBIE*	*PTPN11*	*SMC3*	*TRAF3*
ARPP21	*CD79A*	*DNM2*	*FGFR3*	*IKBKA*	*MDM2*	*NOTCH1*	*RAD21*	*SMC5*	*TRAF6*
ASXL1	*CD79B*	*DNMT1*	*FLI1*	*IKZF1*	*MED12*	*NOTCH2*	*RAD51C*	*SNX7*	*TYK2*
ATF7IP	*CDK4*	*DNMT3A*	*FLT3*	*IKZF2*	*MEF2B*	*NPM1*	*RAG1*	*SOCS1*	*TYK3*
ATM	*CDKN2A*	*DNMT3B*	*FNDC3A*	*IKZF3*	*MEF2C*	*NR3C2*	*RAG2*	*SOX5*	*U2AF1*
ATRX	*CDKN2B*	*EBF1*	*FOXP1*	*IL7R*	*MGA*	*NRAS*	*RASA2*	*SP140*	*U2AF2*
B2M	*CDKN2C*	*ECT2L*	*FYN*	*IRAK1*	*miR125a*	*NSD2*	*RB1*	*SPEN*	*UBR5*
BCL10	*CEBPA*	*EED*	*G6PC3*	*IRAK4*	*miR-142*	*NT5C2*	*REL*	*SPIB*	*USP29*
BCL2	*CEBPE*	*EGR1*	*GAB2*	*IRF1*	*mIR155*	*PAG1*	*RELA*	*SRSF2*	*VPREB1*
BCL6	*CHD2*	*EGR2*	*GATA1*	*IRF4*	*mIR15a*	*PALB2*	*RELB*	*STAG1*	*WHSC1*
BCL7A	*CHK2*	*ELANE*	*GATA2*	*IRF7*	*mIR16-1*	*PAX5*	*RELN*	*STAG2*	*WHSC1L1*
BCOR	*CIITA*	*EP300*	*GATA3*	*ITPKB*	*MIR17HG*	*PDCD1*	*RHOA*	*STAT1*	*WT1*
BCR	*CNOT3*	*EPHA7*	*GCET2*	*JAK1*	*mIR21*	*PDCD1LG2*	*RIPK1*	*STAT3*	*XPO1*
BIRC3	*CREBBP*	*EPOR*	*GFI1B*	*JAK2*	*mir34b*	*PDGFRB*	*ROBO1*	*SUZ12*	*ZAP70*
BLK	*CRLF2*	*ERG*	*GNA13*	*JAK3*	*mir34c*	*PEG3*	*ROR1*	*SYK*	*ZMYM2*
BMI1	*CSF2RA*	*ETV6*	*GNAS*	*JARID2*	*MLL*	*PHF6*	*RPL10*	*TBL1XR1*	*ZMYM3*
BRAF	*CSF3R*	*EZH2*	*GNB1*	*KDM4C*	*MLL2*	*PHIP*	*RPL5*	*TCF3*	*ZRSR2*
BRIP1	*CTBP1*	*FAM46C*	*GPRC5A*	*KDM6A*	*MLL3*	*PIGA*	*RUNX1*	*TERC*	
BTG1	*CTBP2*	*FAM5C*	*HAX1*	*KIT*	*MPL*	*PIK3CA*	*RUNX2*	*TERT*	
BTK	*CTCF*	*FANCA*	*HIST1H1E*	*KLHL6*	*MS4A1*	*PIK3CB*	*SAMHD1*	*TET1*	
BTLA	*CTLA4*	*FANCB*	*HIST1H2AD*	*KRAS*	*MYB*	*PIK3CG*	*SETBP1*	*TET2*	
C22orf194	*CTNNA1*	*FANCC*	*HIST1H2BE*	*LAMB4*	*MYD88*	*PIK3R1*	*SETD2*	*TGDS*	
