# Supplementary material for: Leukemia stemness and co-occurring mutations drive resistance to IDH inhibitors in acute myeloid leukemia
Source: Nat Commun. 2021 May 10;12:2607. doi: 10.1038/s41467-021-22874-x (PMC8110775; doi:10.1038/s41467-021-22874-x)
Supplement: Supplementary file 3 — Description of Additional Supplementary Files [file 41467_2021_22874_MOESM3_ESM.pdf]

Description of Additional Supplementary Files

File Name: Supplementary Data 1
Description: 558 DMP showing a consistent trend of promoter hypermethylation and gene downregulation in Cluster 2.

File Name: Supplementary Data 2
Description: List of 50 amplicons covered by the single cell DNA sequencing.

File Name: Supplementary Data 3
Description: Driver mutation list of 59 baseline samples.
