# Supplementary material for: Leukemia stemness and co-occurring mutations drive resistance to IDH inhibitors in acute myeloid leukemia
Source: Nat Commun. 2021 May 10;12:2607. doi: 10.1038/s41467-021-22874-x (PMC8110775; doi:10.1038/s41467-021-22874-x)
Supplement: Supplementary file 5 — Reporting Summary [file 41467_2021_22874_MOESM5_ESM.pdf]

nature research

Corresponding author(s): Koichi Takahashi
Last updated by author(s): 3/14/2021

Reporting Summary

Nature Research wishes to improve the reproducibility of the work that we publish. This form provides structure for consistency and transparency in reporting. For further information on Nature Research policies, see our Editorial Policies and the Editorial Policy Checklist.

Statistics

For all statistical analyses, confirm that the following items are present in the figure legend, table legend, main text, or Methods section.

n/a	Confirmed	
☐	☒	The exact sample size (n) for each experimental group/condition, given as a discrete number and unit of measurement
☐	☒	A statement on whether measurements were taken from distinct samples or whether the same sample was measured repeatedly
☐	☒	The statistical test(s) used AND whether they are one- or two-sided<br>*Only common tests should be described solely by name; describe more complex techniques in the Methods section.*
☒	☐	A description of all covariates tested
☒	☐	A description of any assumptions or corrections, such as tests of normality and adjustment for multiple comparisons
☐	☒	A full description of the statistical parameters including central tendency (e.g. means) or other basic estimates (e.g. regression coefficient) AND variation (e.g. standard deviation) or associated estimates of uncertainty (e.g. confidence intervals)
☐	☒	For null hypothesis testing, the test statistic (e.g. F, t, r) with confidence intervals, effect sizes, degrees of freedom and P value noted<br>*Give P values as exact values whenever suitable.*
☒	☐	For Bayesian analysis, information on the choice of priors and Markov chain Monte Carlo settings
☒	☐	For hierarchical and complex designs, identification of the appropriate level for tests and full reporting of outcomes
☒	☐	Estimates of effect sizes (e.g. Cohen's d, Pearson's r), indicating how they were calculated

Our web collection on statistics for biologists contains articles on many of the points above.

Software and code

Policy information about availability of computer code

Data collection	No software was used.
Data analysis	R version 3.3.2; DESeq2 version 1.14.1; ChAMP version 2.4.0; Tapestri Pipeline version 1.4.1; mutect version 1.1.4; pindel version 0.2.4; GATK version 3.1; BWA version 0.7.5a; STAR version 2.3.0e; HTSeq-count version 0.6.0; ANNOVAR version 2018-04-16; Picard version 1.112; limma version 3.44.3. The custom codes that support the findings of this study is available in GitHub (DOI: 10.5281/zenodo.4606803).

For manuscripts utilizing custom algorithms or software that are central to the research but not yet described in published literature, software must be made available to editors and reviewers. We strongly encourage code deposition in a community repository (e.g. GitHub). See the Nature Research guidelines for submitting code & software for further information.

Data

Policy information about availability of data

All manuscripts must include a data availability statement. This statement should provide the following information, where applicable:

- Accession codes, unique identifiers, or web links for publicly available datasets
- A list of figures that have associated raw data
- A description of any restrictions on data availability

Raw targeted deep sequencing and single-cell targeted DNA-seq data generated in this study have been deposited in the SRA with the accession number PRJNA713342 (https://www.ncbi.nlm.nih.gov/bioproject/PRJNA713342). Raw methylation and RNA-Seq data generated in this study have been deposited in the GEO with the accession number GSE153349 (https://www.ncbi.nlm.nih.gov/geo/query/acc.cgi?acc=GSE153349). The full list of detected baseline driver mutations was shown in Supplementary Data 3. Source data for all relevant figures and tables are provided with this paper. Public databases used in this study include 1000 Genome Database (http://www.1000genomes.org/), ESP6500 Database (http://evs.gs.washington.edu/EVS/), dbSNP ver.129 (http://www.ncbi.nlm.nih.gov/SNP/), Exome Aggregation Consortium database (http://exac.broadinstitute.org/) and COSMIC database (https://cancer.sanger.ac.uk/cosmic).

Field-specific reporting

Please select the one below that is the best fit for your research. If you are not sure, read the appropriate sections before making your selection.

- [x] Life sciences
- [ ] Behavioural & social sciences
- [ ] Ecological, evolutionary & environmental sciences

For a reference copy of the document with all sections, see nature.com/documents/nr-reporting-summary-flat.pdf

Life sciences study design

All studies must disclose on these points even when the disclosure is negative.

Sample size	We studied 60 patients with relapsed or refractory myeloid malignancies (AML N =55, MDS N = 4, and CMML N =1) who received IDH inhibitor therapy in one of the 2 clinical trials conducted in our institution: NCT01915498 (enasidenib for IDH2 mutated patients) and NCT02074839 (ivosidenib for IDH1 mutated patients). Selection of the studied patients was based on the sample availability in our institution alone.
Data exclusions	We did not exclude any samples or data from the analysis.
Replication	In order to maximize the opportunity for multi-omics analysis with the limited amount of specimens from human patients, technical replicate was not performed in DNA sequencing, RNA sequencing, and methylation analysis. However, by having 60 patients in our cohort with some of them sharing similar mutational, methylational, and transcriptomic profiles, we analyzed the differences between patients sharing the similarity in either one of the molecular profiles, which can be interpreted as biological replicates. However, validation of our findings, particularly the association between stemness score and treatment response to IDH inhibitor, should be validated in independent cohort in future studies. We acknowledged this limitation in the paper.
Randomization	We performed a retrospective molecular correlative analysis on samples collected from 2 clinical trials (NCT01915498 and NCT02074839). Both trials were single-arm trial investigating the efficacy of enasidenib or ivosidenib, respectively. Therefore, patients analyzed in this study were not subject to randomization.
Blinding	Blinding was not performed in the clinical trials as both trials were single-arm studies. However, for the current data analysis, mutation analysis, gene expression analysis, and methylation analysis were performed independently (by F.W.) of clinical data collection (by C.D.D.). Molecular data and clinical data were collected independently at different a timeframe without affecting each other.

Reporting for specific materials, systems and methods

We require information from authors about some types of materials, experimental systems and methods used in many studies. Here, indicate whether each material, system or method listed is relevant to your study. If you are not sure if a list item applies to your research, read the appropriate section before selecting a response.

Materials & experimental systems

n/a	Involved in the study	
x		Antibodies
x		Eukaryotic cell lines
x		Palaeontology and archaeology
x		Animals and other organisms
	x	Human research participants
	x	Clinical data
x		Dual use research of concern

Methods

n/a	Involved in the study	
x		ChIP-seq
x		Flow cytometry
x		MRI-based neuroimaging

Human research participants

Policy information about studies involving human research participants

Population characteristics	This study contains 60 patients with relapsed or refractory myeloid malignancies (AML N =55, MDS N = 4, and CMML N =1) who received IDH inhibitor therapy in one of the 2 clinical trials conducted in our institution: NCT01915498 (enasidenib for IDH2 mutated patients) and NCT02074839 (ivosidenib for IDH1 mutated patients). Thirty-eight (63%) patients were IDH2-mutated, 21 (35%) were IDH1-mutated, and 1 (2%) had both mutations. Thirty-eight (63%) patients were treated with enasidenib and 22 (37%) with ivosidenib. Median age of the cohort is 72 (IQR: 60-77). 42% (N=25) of the patients were female.
Recruitment	We studied 60 patients with relapsed or refractory myeloid malignancies (AML N =55, MDS N = 4, and CMML N =1) who received IDH inhibitor therapy in one of the 2 clinical trials conducted in our institution: NCT01915498 (enasidenib for IDH2 mutated patients) and NCT02074839 (ivosidenib for IDH1 mutated patients). Selection of the studied patients was based on the sample availability in our institution (MD Anderson). Compared with the samples that were not analyzed in this study (due to the lack of sample availability), the studied cohort were older and contained more enasidenib-treated cases. Detailed comparison is listed in Table S1.
Ethics oversight	The study was approved by the MD Anderson IRB under protocol PA12-0305, LAB01-473, 2014-0800, and 2014-0408. Written informed consent for sample collection and analysis was obtained from all patients. The study protocols adhered to the Declaration of Helsinki and were approved by the Institutional Review Board at The University of Texas MD Anderson Cancer Center.

Note that full information on the approval of the study protocol must also be provided in the manuscript.

Clinical data

Policy information about clinical studies

All manuscripts should comply with the ICMJEguidelines for publication of clinical research and a completedCONSORT checklist must be included with all submissions.

Clinical trial registration	NCT01915498; NCT02074839.
Study protocol	Study protocols can be accessed through clinicaltrials.gov
Data collection	Clinical data was collected at MD Anderson Cancer Center from the patients enrolled in the clinical trials between Sep 1, 2014 to Aug 1, 2018.
Outcomes	For molecular correlation, the clinical endpoint was treatment response to IDH inhibitors. We used p values estimaed from odds ratio, multilogistic regression analysis and ROC curve analysis for statistical testing between treatment response and molecular features.
